# Supplementary figures and images for: Decoding protein phosphorylation during oocyte meiotic divisions using phosphoproteomics
Source: eLife. 2025 Jul 17;13:RP104255. doi: 10.7554/eLife.104255 (PMC12270485; doi:10.7554/eLife.104255)

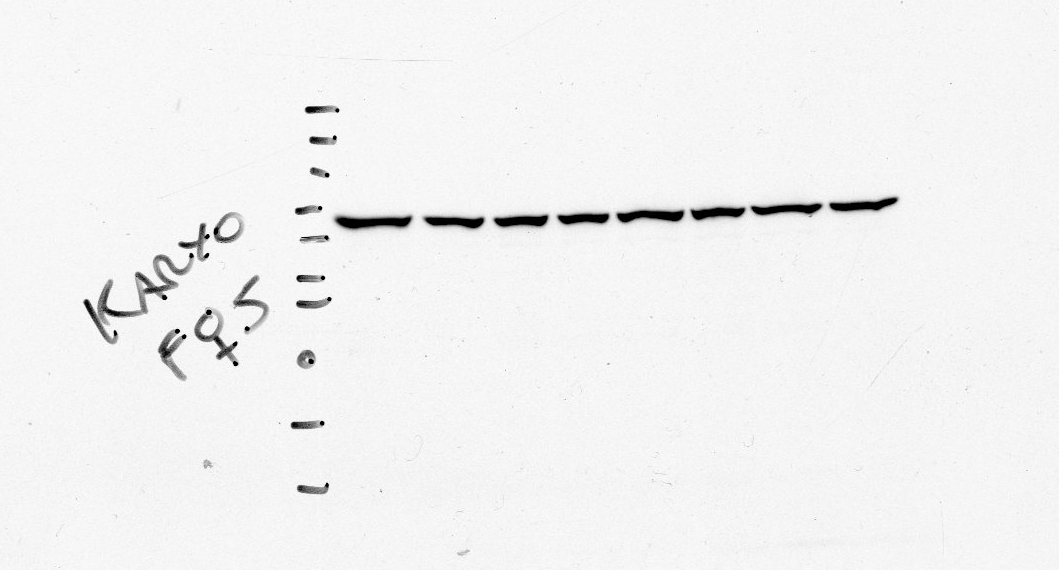

Supplement: Figure 10—source data 1. [file elife-104255-fig10-data1.zip › Figure 9-source data 1/img20250403_16303962.jpg]

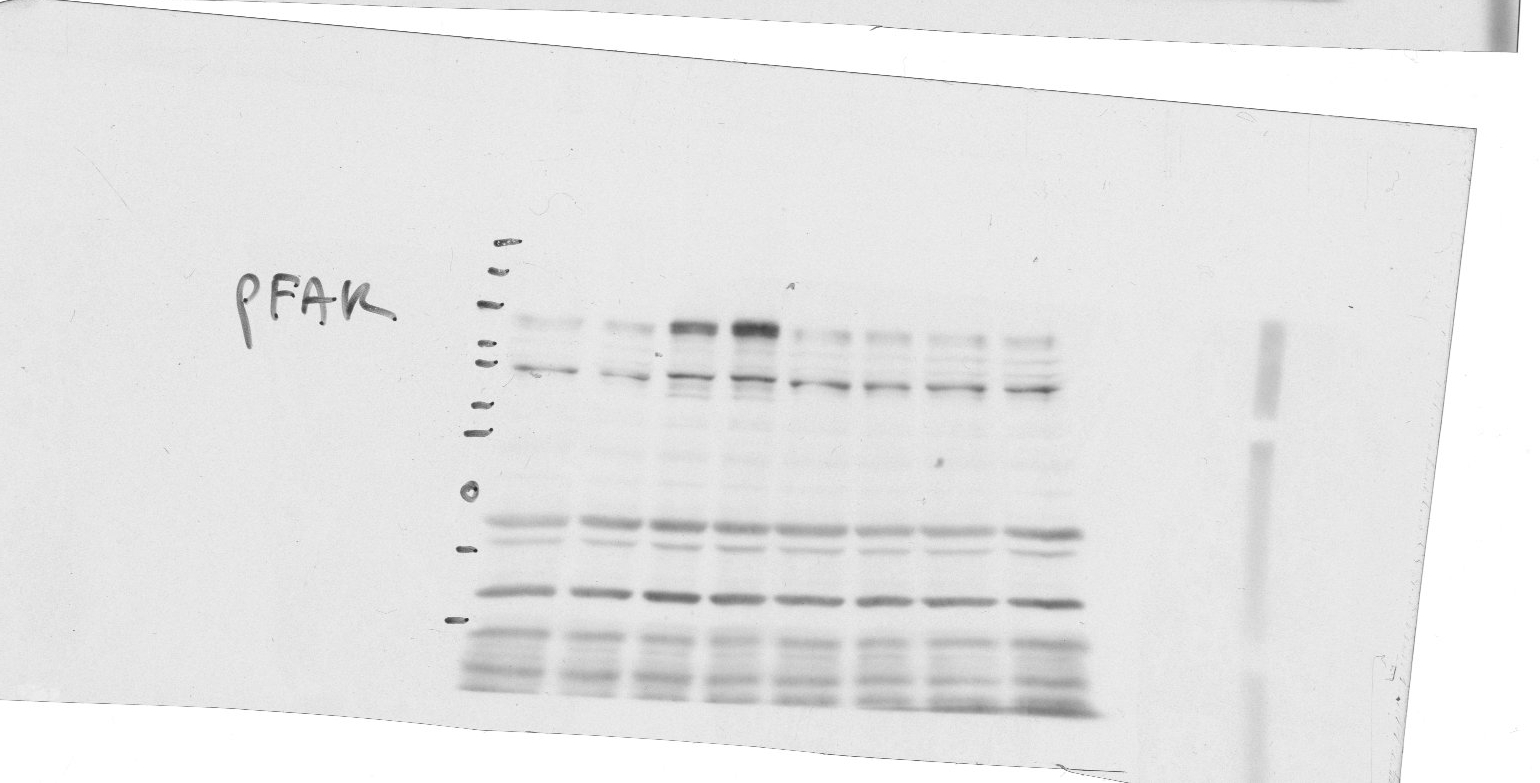

Supplement: Figure 10—source data 1. [file elife-104255-fig10-data1.zip › Figure 9-source data 1/img20250402_16093906.jpg]

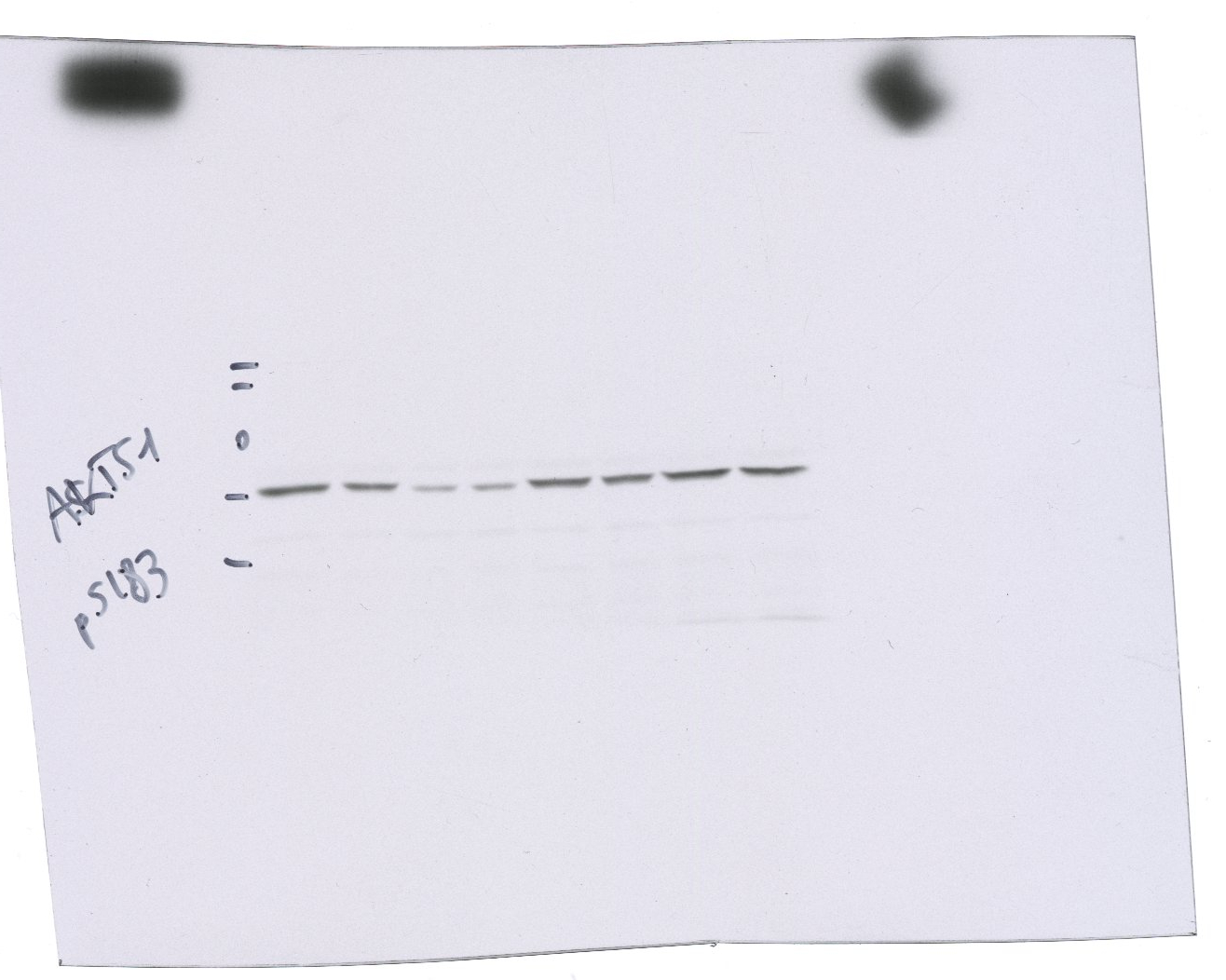

Supplement: Figure 10—source data 1. [file elife-104255-fig10-data1.zip › Figure 9-source data 1/img20250425_16542643.jpg]

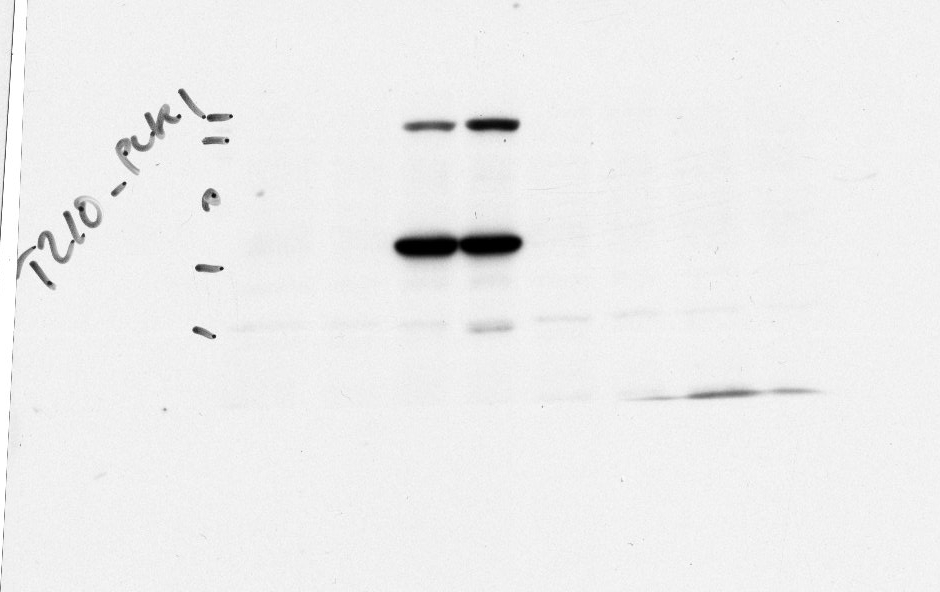

Supplement: Figure 10—source data 1. [file elife-104255-fig10-data1.zip › Figure 9-source data 1/img20250409_17464221.jpg]

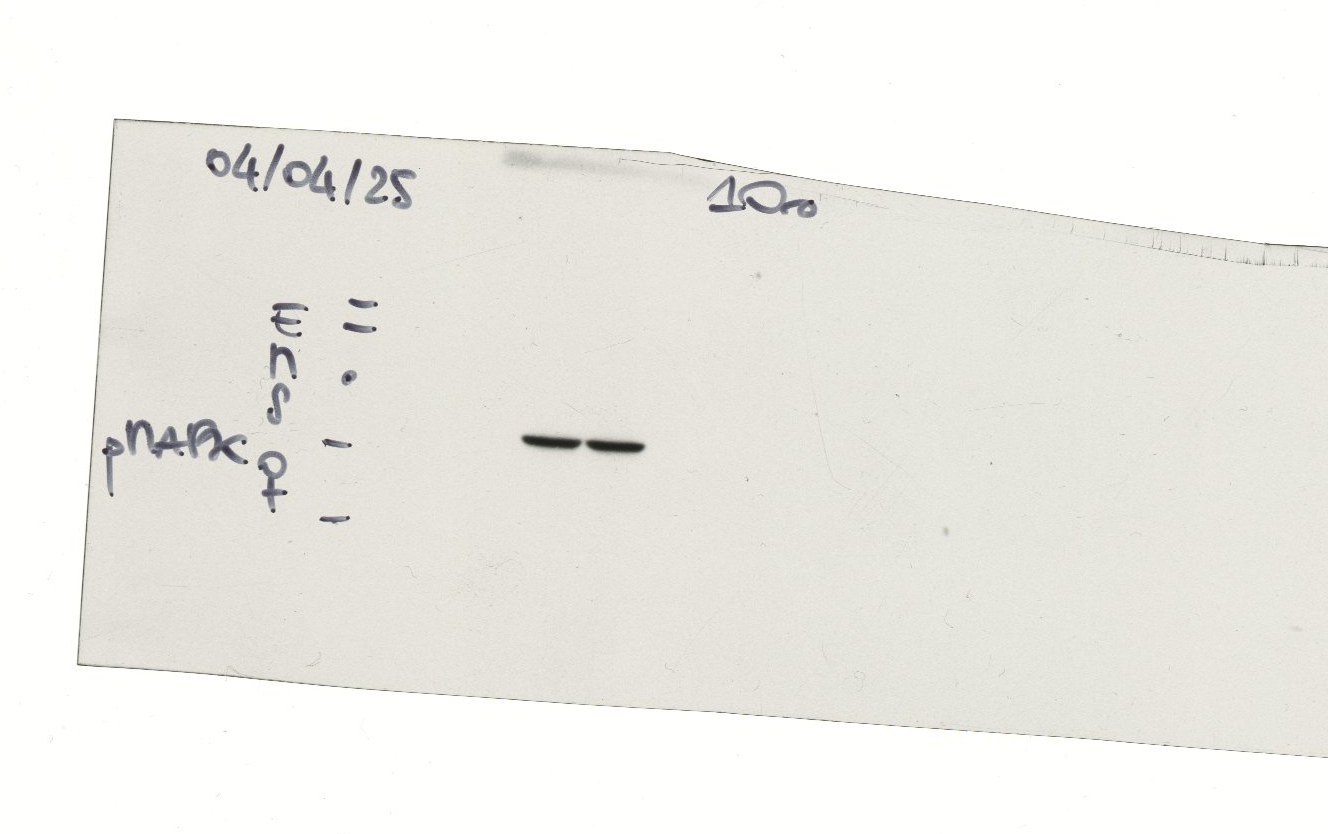

Supplement: Figure 10—source data 1. [file elife-104255-fig10-data1.zip › Figure 9-source data 1/img20250620_10175420.jpg]
