# Supplementary material for: Decoding protein phosphorylation during oocyte meiotic divisions using phosphoproteomics
Source: eLife. 2025 Jul 17;13:RP104255. doi: 10.7554/eLife.104255 (PMC12270485; doi:10.7554/eLife.104255)
Supplement: Figure 10—source data 2. [file blots marked.pdf › Figure 9-source data 2/blots marked.pdf]

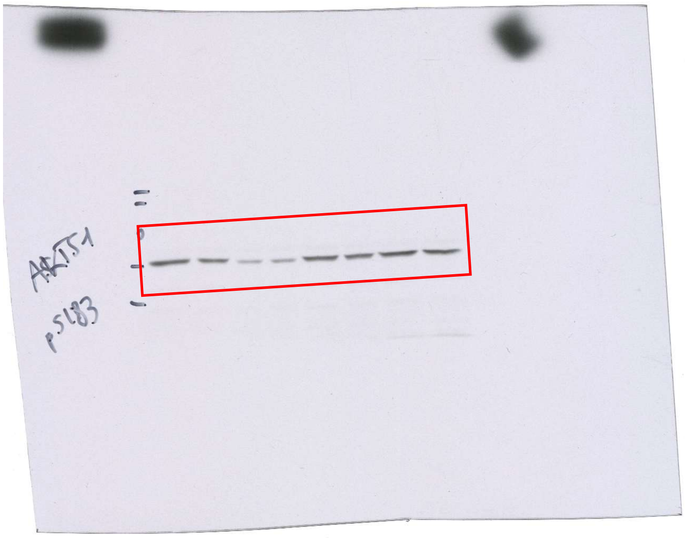
AKTS1
pS183

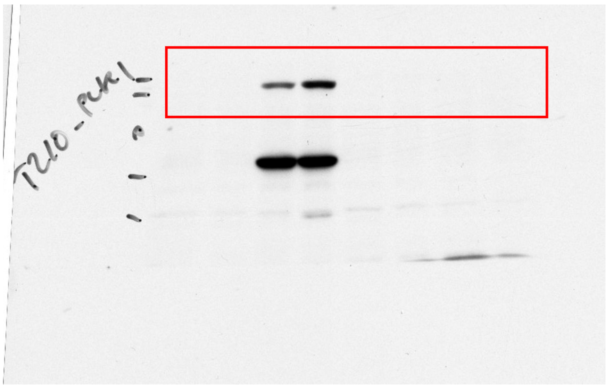
T210_PLK1

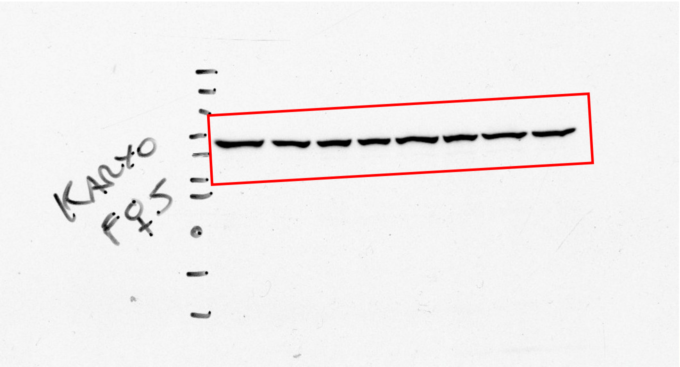
KARYO

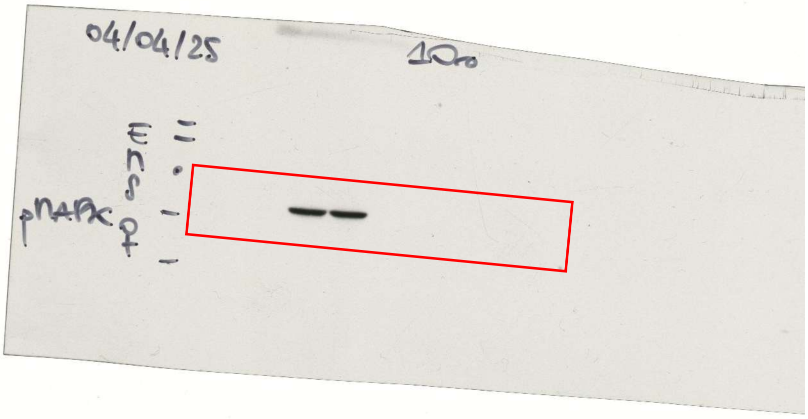
04/04/25
pMAPK

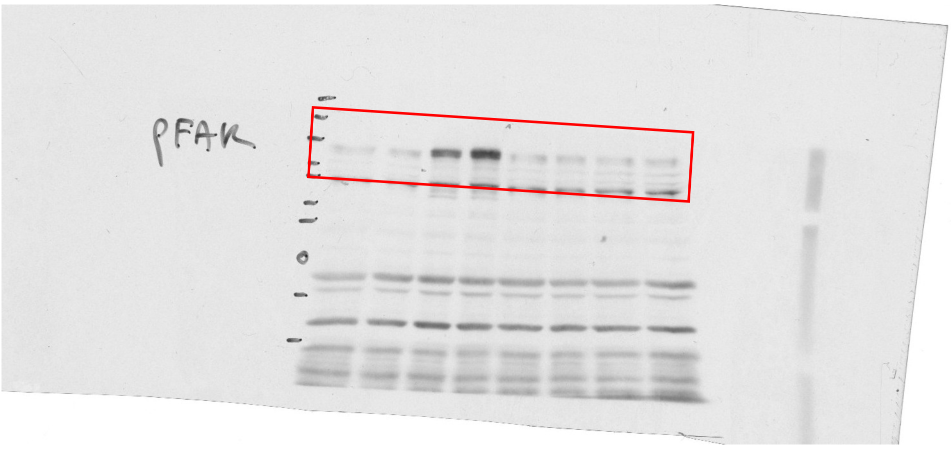
pFAK
